# CT Body Composition Changes Predict Survival in Immunotherapy-Treated Cancer Patients: A Retrospective Cohort Study

**DOI:** 10.3390/cancers18020341

**Published:** 2026-01-21

**Authors:** Shlomit Tamir, Hilla Vardi Behar, Ronen Tal, Ruthy Tal Jasper, Mor Armoni, Hadar Pratt Aloni, Rotem Iris Orad, Hillary Voet, Eli Atar, Ahuva Grubstein, Salomon M. Stemmer, Gal Markel

**Affiliations:** 1 Radiology Department, Rabin Medical Center-Beilinson Hospital, Petah Tikva 49100, Israel; shlomitt@clalit.org.il (S.T.); morar1@clalit.org.il (M.A.); elia@clalit.org.il (E.A.); ahuvag@clalit.org.il (A.G.); 2 Gray Faculty of Medicine and Health Sciences, Tel Aviv University, Tel Aviv 69978, Israel; rotemorad@mail.tau.ac.il; 3 Davidoff Cancer Center and Samueli Integrative Cancer Pioneering Institute, Rabin Medical Center-Beilinson Hospital, Petah Tikva 49100, Israel; hillavar@clalit.org.il (H.V.B.); ronental@clalit.org.il (R.T.); ruthtal@clalit.org.il (R.T.J.); hadarpr1@clalit.org.il (H.P.A.); galma4@clalit.org.il (G.M.); 4 Department of Environmental Economics and Management, Hebrew University of Jerusalem, Rehovot 7610001, Israel; hillary.voet@mail.huji.ac.il; 5 Department of Clinical Microbiology and Immunology, Gray Faculty of Medicine and Health Sciences, Tel Aviv University, Tel Aviv 69978, Israel

**Keywords:** body composition, computed tomography, sarcopenia, skeletal muscle, subcutaneous fat, immunotherapy, longitudinal change, prognosis, deep learning, solid tumors

## Abstract

Background: Computed tomography (CT)-derived body composition parameters, including skeletal muscle and fat indices, are prognosticators in oncology. Most studies focus on baseline body-composition parameters; however, changes during treatment may provide better prognostic value. Standardized methods for measuring/reporting these parameters remain limited. Methods: This retrospective study included patients who were treated with immunotherapy for non-small cell lung cancer (NSCLC), renal cell carcinoma (RCC), or melanoma between 2017 and 2024 and had technically adequate baseline and follow-up CT scans. Body composition was analyzed using a novel, fully automated software (CompoCT) for L3 slice selection and segmentation. Body composition indices (e.g., skeletal muscle index [SMI]) were calculated by dividing the cross-sectional area by the patient’s height squared. Results: The cohort included 376 patients (mean [SD] age 66.4 [11.4] years, 67.3% male, 72.6% NSCLC, 14.6% RCC, and 12.8% melanoma). During a median follow-up of 21 months, 220 (58.5%) died. Baseline body composition parameters were not associated with mortality, except for a weak protective effect of higher SMI (HR = 0.98, *p* = 0.043). In contrast, longitudinal decreases were strongly associated with increased mortality. Relative decreases in SMI (HR, 1.17; 95% CI, 1.07–1.27) or subcutaneous fat index (SFI) (HR, 1.11; 95% CI, 1.07–1.15) significantly increased mortality risk. Multivariate models showed similar concordance (0.65) and identified older age, NSCLC tumor type, and relative decreases in SMI and SFI (per 5% units) as independent predictors of mortality. Conclusions: Longitudinal decreases in skeletal muscle and subcutaneous fat were independent predictors of mortality in immunotherapy-treated patients. Automated CT-based body composition analysis may support treatment decisions during immunotherapy.

## Simple Summary

Body composition indices related to skeletal muscle and body fat can be derived from computed tomography (CT) imaging. However, longitudinal changes in these indices during the course of cancer treatment may be better predictors of outcomes than these indices at baseline. Here, we used a novel, fully automated software (CompoCT) to measure composition indices from CT imaging and showed that in patients treated with immunotherapy for non-small cell lung cancer, renal cell carcinoma, or melanoma, longitudinal decreases in skeletal muscle and subcutaneous fat were strong independent predictors of death, with prognostic value exceeding that of baseline measures. Moreover, these indices remained statistically significant after adjusting for age, sex, and tumor type. Therefore, automated CT-based body composition analysis may enhance objective risk stratification and support treatment decisions during immunotherapy. Furthermore, the integration of such automated imaging tools may also support metabolic research by providing standardized quantitative markers of body composition dynamics.

## 1. Introduction

Immune checkpoint inhibitors (ICIs) have transformed the treatment landscape of several advanced solid malignancies, leading to substantial improvements in overall survival for select patient subgroups. Nevertheless, a considerable proportion of patients derive limited benefit, and mortality rates remain high despite treatment. Early identification of patients with increased risk of death is crucial for optimizing treatment strategies, supportive care, and clinical decision-making. Current clinical and laboratory markers offer limited prognostic accuracy, particularly early during treatment, highlighting the need for complementary tools that can be readily derived from existing clinical data, such as computed tomography (CT) imaging. In this context, CT-derived body composition parameters have emerged as promising prognostic biomarkers. The prognostic value of CT-derived body composition parameters at baseline has been well established across various solid cancers treated with immunotherapy, including non-small cell lung cancer (NSCLC), melanoma, and renal cell carcinoma (RCC) [1,2,3,4,5,6,7,8,9]. Baseline skeletal muscle and fat indices are associated with survival and treatment outcomes, but static measurements alone do not adequately capture dynamic metabolic changes during therapy. Emerging evidence suggests that longitudinal changes in body composition provide more robust predictive value [2,10,11,12,13,14,15].

Notably, Chaunzwa et al. recently demonstrated in the largest published cohort of NSCLC patients that longitudinal muscle loss during immunotherapy was more strongly associated with overall survival than baseline muscle mass [2]. This finding aligns with smaller studies reporting that the prognostic value of longitudinal measures is superior to those of baseline measures across tumor types. For example, Willemsen et al. described body composition dynamics predicting outcomes in head and neck cancer [10], Degens et al. reported early muscle loss as a prognostic marker in NSCLC [11], and Crombé et al. linked early decreases in muscle and fat with progression-free survival in metastatic cancers, though their focus was disease progression rather than mortality [12]. Jin et al. showed that longitudinal body composition trajectories were associated with worse response and decreased survival in patients with hepatocellular carcinoma [16], although no comparison was made to baseline predictive value. Loosen et al. showed that progressive sarcopenia predicted poor outcomes in a small cohort of patients with a variety of tumors, yet adipose tissue was not evaluated [17]. These earlier investigations had limited generalizability due to a single tumor type focus, small sample sizes, or the absence of multivariable analysis. In addition, most prior studies relied on manual or semi-automated methods, which are time-consuming and limit the feasibility of applying longitudinal body composition analysis in clinical practice. Heterogeneity in segmentation techniques and reporting conventions further hinders reproducibility and clinical adoption [18,19,20,21]. Addressing these gaps requires standardized evaluation of both relative and absolute longitudinal changes in CT-derived skeletal muscle and adipose tissue, assessed in a large, multi-tumor cohort.

In recent years, fully automated deep learning algorithms trained to derive body composition parameters from CT scans have been developed [22,23,24,25]. These algorithms have been validated, and their utility for routine diagnostic applications has been demonstrated [2,6,26,27,28]. Automation enables high-throughput, standardized, and reproducible extraction of body composition metrics, facilitating longitudinal tracking across large patient cohorts without additional imaging or manual effort. Such tools offer an opportunity to integrate opportunistic body composition analysis into routine oncologic workflows, supporting dynamic risk stratification.

In this study, we leveraged a novel, fully automated deep learning pipeline to analyze CT-derived body composition parameters at baseline and during treatment in a large, heterogeneous cohort of patients with metastatic NSCLC, RCC, or melanoma receiving ICIs. We aimed to evaluate whether longitudinal changes in skeletal muscle and adipose tissue provide prognostic information beyond that gained from baseline measurements and to explore their potential for standardized, automated risk stratification during immunotherapy. To our knowledge, this is the first large, multi-tumor study to evaluate longitudinal changes in multiple CT-derived body composition compartments using a fully automated deep learning tool during immunotherapy, and to assess their prognostic value for overall survival.

## 2. Materials and Methods

### 2.1. Study Population

This single-center retrospective cohort study included all consecutive adult patients (≥18 years) who received immunotherapy (ICIs) for metastatic NSCLC, RCC, or melanoma at Rabin Medical Center (RMC) between January 2017 and November 2024. Inclusion criteria included availability of CT scans of sufficient diagnostic quality, at both baseline and follow-up. Baseline CT was defined as a scan performed within the 90 days preceding or up to 30 days following the first day of treatment. Follow-up CT was defined as a scan performed between 40 and 120 days after the first day of ICI treatment. Patients were excluded if they had multiple active primary malignancies, their CT scans at either baseline or follow-up were inadequate, or if no data were available regarding height or ICI treatment dates. Multiple active malignancies were defined as either a history of metastatic disease (M1) diagnosed at any time before the index malignancy (NSCLC, RCC, or melanoma), or a diagnosis of a separate malignancy (excluding non-melanoma skin cancer) within 12 months prior to the index cancer diagnosis. ICI medications included nivolumab, pembrolizumab, atezolizumab, ipilimumab, and durvalumab. CT scans were considered inadequate for the study if the image quality was substantially degraded, the L3 vertebral level was not visualized, or if body composition segmentation could not be performed due to technical limitations such as metal artifacts, incomplete visualization of the abdominal circumference, or patient positioning (e.g., arms adjacent to the abdomen) that compromised image quality.

### 2.2. Data Collection

Demographic and clinical data, including height, were extracted from the anonymized electronic medical records of Clalit Health Services (MDClone, Beer Sheba, Israel). CT images were retrieved from the picture archiving and communication system of the Clalit Health Services. Vital status and date of death were obtained from institutional medical records and national registries. Patients with missing clinical or imaging data required for analysis were excluded.

### 2.3. Body Composition Measurement

Body composition was assessed using CompoCT (version 1), a fully automated software developed at the Samueli Integrative Cancer Pioneering Institute, RMC. The model was trained and validated on independent cohorts of patients scanned at RMC. Training was performed on CT scans of emergency department patients, and validation was performed on CT scans of oncological patients. Details of the model architecture, training, and validation are provided in the Supplementary Information (Supplementary Material; Figures S1 and S2).

**Figure 1:**
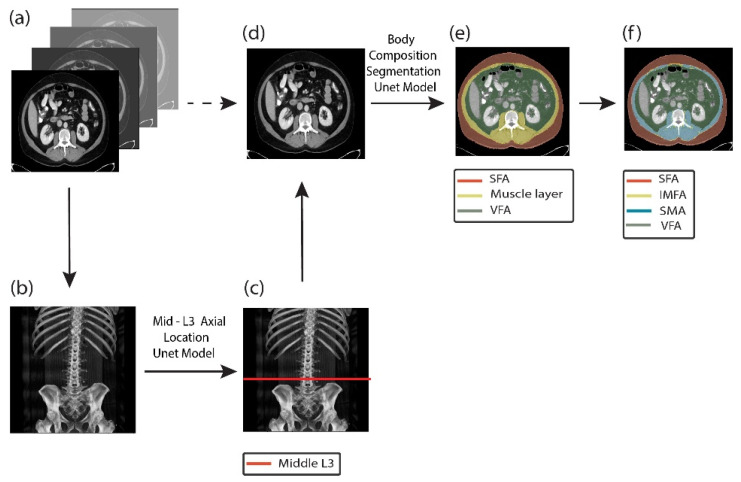
Automated pipeline for L3 vertebral slice selection and body composition segmentation. (**a**) Input consists of 3D abdomen CT scan. (**b**) A 2D coronal Maximum Intensity Projection (MIP) is generated from the 3D scan to enhance anatomical visualization. (**c**) A U-Net-based segmentation model is applied on the coronal MIP to automatically detect and localize the L3 vertebral level. (**d**) Based on the predicted L3 location, the middle axial slice at the L3 level is extracted from the original 3D CT scan. (**e**) The selected L3 slice is processed by a segmentation model to delineate key body composition compartments: subcutaneous adipose tissue (SCA, red), skeletal muscle layer (Muscle, yellow), and visceral fat area (VFA, green). (**f**) Hounsfield Unit (HU) thresholds are then applied to further classify the segmented muscle region into skeletal muscle area (SMA, blue) and intermuscular fat area (IMFI, yellow).

The overall workflow for automated body composition analysis using CompoCT is illustrated in Figure 1. A single axial slice at the L3 vertebral level was automatically selected by a deep learning model. Skeletal muscle, subcutaneous adipose tissue, visceral adipose tissue, and intermuscular fat were segmented automatically, and cross-sectional areas were normalized for patient height squared (m^2^) to derive the skeletal muscle index (SMI), subcutaneous fat index (SFI), visceral fat index (VFI), and intermuscular fat index (IMFI). For tissue classification, Hounsfield unit (HU) thresholds were applied to the segmented compartments. Skeletal muscle was defined using an attenuation range of −29 to +150 HU, and adipose tissue compartments were defined using −190 to −30 HU, consistent with the established CT-based body composition literature.

The L3 localization model was trained on 828 CT scans and validated on 172 scans. The tissue segmentation model was trained on 620 scans and validated on 125 scans. Both models demonstrated robust generalization to independent oncologic cohorts. In the validation sets, CompoCT had mean Dice similarity coefficients exceeding 92.8% across all relevant tissue compartments, and 94.2% accuracy in localization of mid-L3 vertebral height, within a predefined absolute error below 10 mm (Supplementary Material, Figures S1 and S2).

For this cohort of immunotherapy-treated patients with solid tumors, all mid-L3 slice selections and tissue segmentations underwent visual validation by an abdominal radiologist with 17 years of experience. L3 localization errors were identified in 5.7% of cases and were manually corrected to preserve cohort size. Segmentation errors were observed in 6.2% of cases and were excluded from the final analysis. The CT scans analyzed in this study were acquired during routine clinical care using heterogeneous imaging protocols, including both contrast-enhanced and non–contrast-enhanced examinations. As intravenous contrast administration affects tissue attenuation values, the analyses were based on segmentation-derived cross-sectional areas rather than attenuation-based muscle quality metrics. Accordingly, subclassification of muscle by density was not included in statistical models.

### 2.4. Statistical Analysis

Statistical analyses were conducted using SPSS (v28.0.0) and Python (v3.9). Descriptive statistics, including mean, standard deviation (SD), median, and interquartile range, were used to summarize patient demographics and body composition metrics, as appropriate.

Overall survival was defined as the time from initiation of immunotherapy to death from any cause. Patients who were alive at the last follow-up were censored at that date. For survival analysis, Cox proportional hazards regression models were applied to evaluate associations between body composition metrics and overall survival. Univariate models were constructed, with hazard ratios (HRs), 95% confidence intervals (CIs), and *p*-values reported. Survival analyses were performed using the lifelines and statsmodels packages in Python. *p*-value < 0.05 was considered statistically significant, unless stated otherwise. Body composition variables were analyzed both as continuous measures (per unit increase) and as relative percentage change from baseline.

Multivariable Cox regression models included pre-specified clinical variables (age, sex, and tumor type). To quantify the added prognostic value of longitudinal CT-derived body composition metrics beyond standard clinical predictors, we subsequently evaluated imaging biomarkers for inclusion in extended models and retained those demonstrating independent prognostic value while avoiding multicollinearity. Model performance was assessed using concordance indices (C-index), and improvement in discrimination between clinical and extended models was quantified. To assess robustness to potential clinical confounding, an expanded multivariable Cox regression model incorporating the Eastern Cooperative Oncology Group (ECOG) performance status, treatment regimen category (single-agent vs. combination), and available baseline laboratory markers (albumin and neutrophil-to-lymphocyte ratio [NLR]) was evaluated in a subset of patients with available data.

Sensitivity analyses were performed to evaluate the robustness of the findings and address potential sources of bias. These analyses included (1) assessment of potential contamination of baseline measurements by early treatment-related changes through examination of the distribution of the intervals between baseline CT acquisition and treatment initiation, as well as by performing analyses restricting baseline CT scans to the pre-treatment period only; (2) addressing the variability in the interval between baseline and follow-up CT scans, via normalizing longitudinal body composition changes by scan interval, expressing them as relative percentage change per 30 days, and repeating the Cox regression models using these time-normalized variables; (3) testing the robustness of the ≥5% relative decline threshold used to define body composition decrease by performing additional Kaplan–Meier analyses with alternative cutoffs of ≥3% and ≥10%; and (4) correcting for potential immortal-time bias introduced by requiring availability of a follow-up CT scan, by performing a landmark analysis in which overall survival was recalculated from the date of the follow-up CT scan, and repeating the Cox regression models using survival time from this landmark.

### 2.5. Ethics Statement

The study was approved by the institutional review board of RMC and was granted a waiver for obtaining informed consent due to its retrospective design. The study was conducted in accordance with the declaration of Helsinki.

## 3. Results

### 3.1. Study Patients

**Figure 2:**
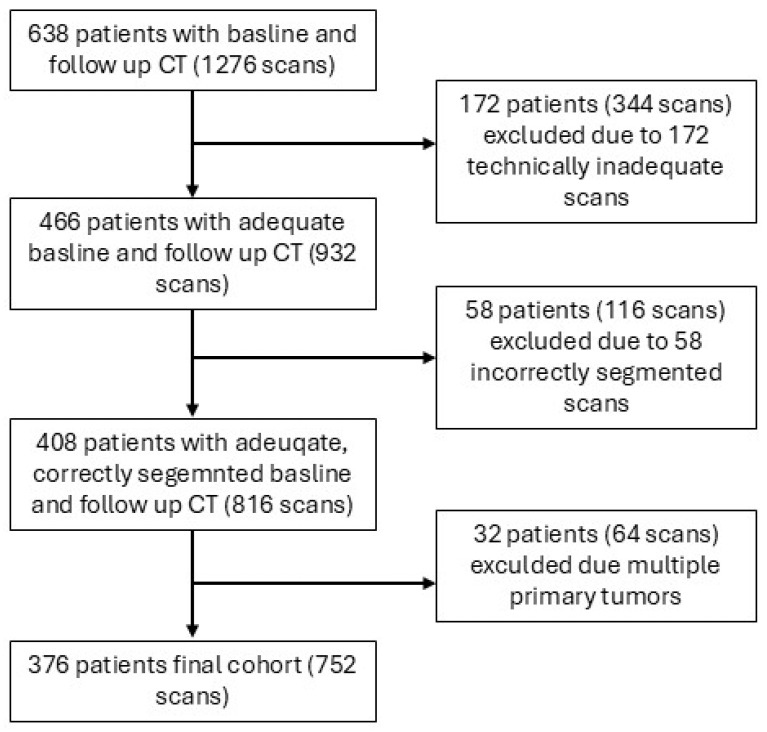
Patient selection flowchart. CT, Computed Tomography.

A total of 638 patients and 1276 corresponding scans (i.e., 2 scans per patient, one at baseline and one at follow-up) were initially identified. These were patients with metastatic NSLC, RCC, or melanoma, who received ICI during the study period and had baseline and follow-up CT scans. Upon evaluation of the scans, 172 scans from 172 patients were found to be inadequate (incomplete abdominal coverage, severe motion or streak artifacts, metal artifacts, missing image series, or non-axial reconstructions unsuitable for L3 analysis), leading to the exclusion of 172 patients (i.e., 344 corresponding scans). Of the remaining 466 patients (932 scans), 58 scans from 58 patients had segmentation errors in the scans that could not be manually corrected, leading to the exclusion of 58 patients (i.e., 116 corresponding scans). In addition, L3 localization errors were detected in a subset of scans but were successfully corrected manually and did not lead to patient exclusion. Lastly, of the remaining 408 patients (816 scans), 32 patients (64 scans) were excluded due to the patients having multiple primary malignancies. Thus, the final cohort consisted of 376 patients with 752 corresponding scans (Figure 2). The characteristics of the cohort, including demographics, treatment regimens, follow-up time, and interval between CT scans, overall and by tumor type, are presented in Table 1. The majority of the cohort were males (67.3%), the mean (SD) age for the entire cohort was 66.4 (11.4) years, and the most common treatment regimen was pembrolizumab monotherapy (58.5%). Of the 376 patients, 273 (72.6%) had NSCLC, 55 (14.6%) had RCC, and 48 (12.8%) had melanoma. Patients with RCC were younger on average, although the difference in age across tumor types was not statistically significant. ICI regimen distribution differed significantly between tumor types (Table 1). The median (range) interval between the baseline and follow-up CT scan was 100 (40–199) days for the entire cohort. This interval differed significantly between tumor types, with the shortest interval observed among NSCLC patients (median [range], 98 [40–191] days).

**Figure 3:**
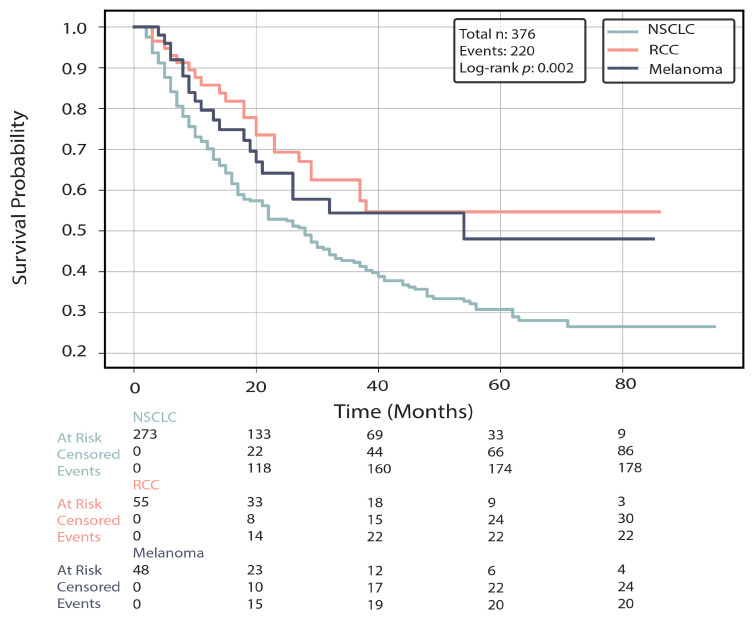
Kaplan–Meier overall survival curves stratified by diagnosis type. Survival was measured from the first day of immune checkpoint inhibitor (ICI) treatment. The log-rank test was calculated from all data evaluating the statistical significance of the difference in overall survival between the 3 diagnosis types. The number of patients at risk, censored, and events are listed below the time axis for each group.

The median (range) follow-up time from the initiation of the ICI treatment was 21 (2–95) months for the entire cohort and 20 (2–95), 29 (3–86), and 20 (4–85) months for the NSCLC, RCC, and melanoma groups, respectively. Overall, 220 (58.5%) deaths were reported. Mortality rates varied by tumor type, being highest among patients with NSCLC (178/273, 65.2%), followed by those with RCC (22/55, 40.0%) and melanoma (20/48, 41.7%) (Table 1, Figure 3).

### 3.2. CT-Derived Body Composition Parameters

The distribution of CT-derived body composition parameters at baseline and follow-up, alongside the change during treatment, are presented in the Supplementary Material (Table S1). Associations were generally more evident at follow-up and for relative percentage changes than at baseline, particularly across fat compartments, with the strongest associations observed between decreases in IMFI and VFI (r = 0.69, *p* < 0.001), between IMFI and SFI at baseline and follow-up (r = 0.61), and decreases in SFI and VFI (r = 0.59). In contrast, SMI was less strongly correlated with fat compartments, indicating that muscle and fat reflect partly distinct domains.

### 3.3. CT-Derived Body Composition Parameters and Correlation with Mortality

**Figure 4:**
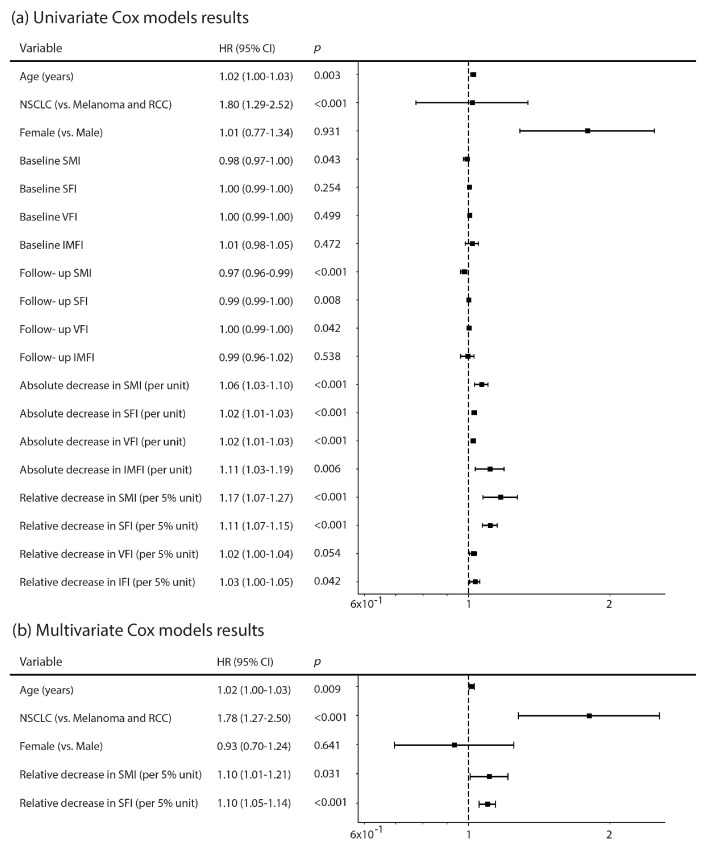
Univariate (**a**) and multivariate (**b**) analyses (Cox model results).

Baseline CT-derived body composition parameters were not significantly associated with mortality, aside from a weak protective effect of higher baseline SMI (HR, 0.98; 95% CI, 0.97–1.00; *p* = 0.043). In contrast, for several follow-up CT-derived BC measures, higher values were associated with lower mortality risk, including SMI (HR, 0.97; 95% CI, 0.96–0.99; *p* < 0.001) and SFI (HR, 0.99; 95% CI, 0.99–1.00; *p* = 0.008). Univariate analysis results are shown in Figure 4a.

With respect to change over time, absolute decreases in all 4 compartments, SMI (HR, 1.06; 95% CI, 1.03–1.10; *p* < 0.001), SFI (HR, 1.02; 95% CI, 1.01–1.03; *p* < 0.001), VFI (HR, 1.02; 95% CI, 1.01–1.03; *p* < 0.001), and IMFI (HR, 1.11; 95% CI, 1.03–1.19; *p* = 0.006), were significantly associated with increased mortality. Similarly, relative decreases in SMI (HR, 1.17; 95% CI, 1.07–1.27; *p* < 0.001), SFI (HR, 1.11; 95% CI, 1.07–1.15; *p* < 0.001), and IMFI (HR, 1.03; 95% CI, 1.00–1.05; *p* = 0.042), were significantly associated with increased mortality, whereas the association with VFI (HR, 1.02; 95% CI, 1.00–1.05; *p* = 0.054) did not reach statistical significance. HR associated with relative decreases in body composition measures were calculated per 5% change, a threshold selected a priori based on available sarcopenia literature and in order to enhance clinical interpretability.

**Figure 5:**
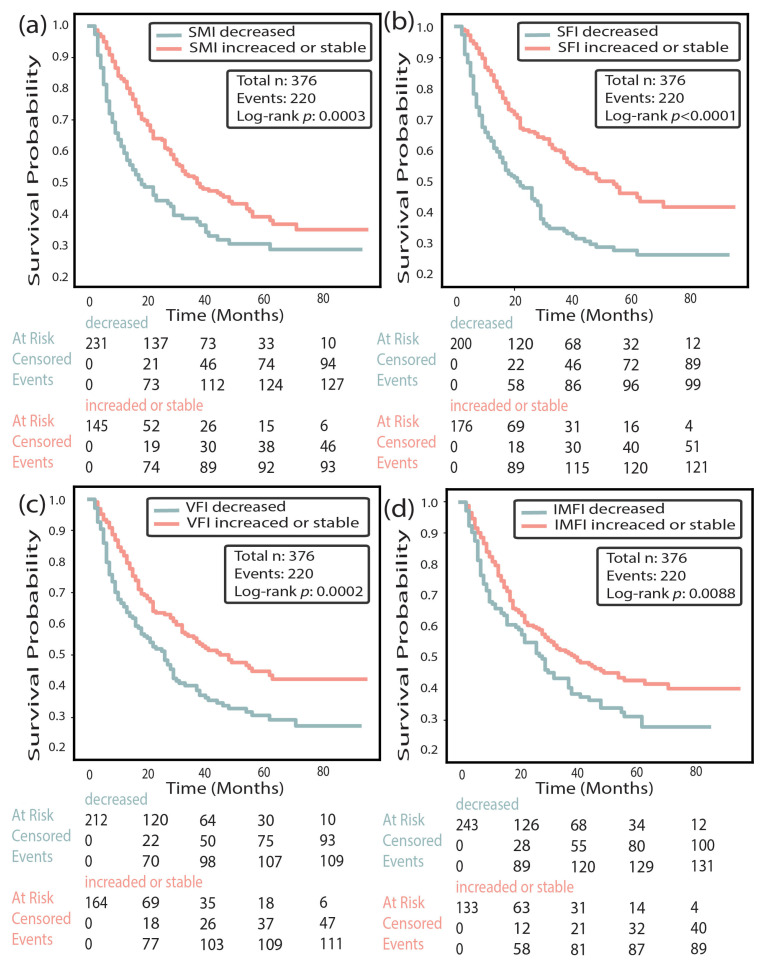
Kaplan–Meier overall survival analysis based on relative changes in body composition indices. Overall survival was stratified by relative changes in (**a**) skeletal muscle index (SMI), (**b**) subcutaneous fat index (SFI), (**c**) visceral fat index (VFI), and (**d**) intermuscular fat index (IMFI) between baseline and follow-up CT scans. Decreasing was defined as more than 5% relative decrease in the relevant body component. Log-rank test was calculated from all data; the number of patients at risk, censored, and events are displayed below each plot.

Kaplan–Meier analyses demonstrated that patients with ≥5% decreases in SMI, SFI, or IMFI had significantly shorter overall survival compared to those with stable or increased values (*p* < 0.001 for all; Figure 5). For VFI, the difference was borderline significant (*p* = 0.046) (Figure 5). Mean overall survival was 17 vs. 36 months for patients with decreased vs. stable/increased SMI, 18 vs. 45 months for SFI, 26 vs. 33 months for IMFI, and 22 vs. 38 months for VFI.

Multivariable Cox regression models including age, sex, and tumor type identified longitudinal decreases in skeletal muscle and subcutaneous fat indices as independent predictors of mortality (Table 2, Figure 4b). Older age and NSCLC tumor type were consistently associated with an increased mortality risk. Among CT-derived body composition metrics, relative changes in SMI and SFI showed the most robust independent associations with survival and were therefore included in the extended prognostic model, with relative rather than absolute changes prioritized to improve interpretability across body sizes and baseline distributions. A clinical core model including age, sex, and tumor type demonstrated modest discrimination for overall survival (C-index = 0.58). Addition of longitudinal body composition changes (relative SMI and SFI decreases) improved model performance (C-index = 0.65), corresponding to an absolute improvement in discrimination of 0.07. In the extended model, relative decreases in SFI (per 5% unit; HR = 1.10, 95% CI, 1.05–1.14, *p* < 0.001) and SMI (per 5% unit; HR = 1.10, 95% CI, 1.01–1.21, *p* = 0.03) remained independent predictors of mortality.

To assess robustness to potential clinical confounding, an expanded multivariable Cox model incorporating ECOG performance status, treatment regimen category, and baseline laboratory markers was evaluated in a subset analysis (Supplementary Material, Table S2). In this model, relative decreases in SFI remained independently associated with mortality, whereas relative decreases in SMI showed consistent effect magnitude but did not reach formal statistical significance in the smaller subset. Tumor type similarly showed consistent effect magnitude but did not reach statistical significance in this expanded model.

### 3.4. Sensitivity Analyses

Baseline CT scans were obtained at variable times relative to treatment initiation (−90 to +30 days). Most of these scans were acquired before treatment initiation (Supplementary Material, Figure S3). Restricting baseline CTs to pre-treatment scans did not substantially change the associations between longitudinal decreases in skeletal muscle and subcutaneous fat indices and mortality (Supplementary Material, Figure S4). Due to the variation in the interval between the baseline and follow-up scans (40–120 days), additional analyses using scan-interval–normalized changes (% per 30 days) were performed. These analyses demonstrated that time-normalized decreases in skeletal muscle and subcutaneous fat indices remained significantly associated with mortality (Supplementary Material, Figure S5). Additionally, using alternative decline thresholds (≥3% and ≥10%) yielded consistent separation of survival curves across all body composition compartments (Supplementary Material, Figures S6 and S7).

Finally, in landmark analyses correcting for potential immortal-time bias, relative decreases in skeletal muscle index (per 5% unit; HR = 1.17, 95% CI, 1.07–1.27, *p* < 0.001) and subcutaneous fat index (per 5% unit; HR = 1.11, 95% CI, 1.07–1.15, *p* < 0.001) remained independent predictors of mortality, with effect sizes comparable to the primary analysis (Supplementary Material, Figures S8 and S9).

## 4. Discussion

This study showed that longitudinal changes in CT-derived body composition parameters during treatment with ICIs in patients with various solid tumors were strongly associated with survival outcomes. Baseline parameters showed only a weak association with mortality limited to SMI, whereas follow-up measurements and, most notably, absolute and relative reductions during treatment demonstrated stronger prognostic value. In the multivariate analysis, older age, NSCLC tumor type, and relative decreases in SMI and SFI (per 5% units) were independently associated with increased mortality risk. To our knowledge, this is the first large, multi-tumor cohort study to evaluate longitudinal changes in multiple CT-derived body composition compartments using fully automated deep learning algorithms during immunotherapy and to demonstrate their independent prognostic value for overall survival.

While baseline CT-derived body composition parameters have been established as prognostic biomarkers in prior studies [1,2,3,4,5,6,7,8,9], their effect sizes in our study were modest (e.g., baseline SMI HR of 0.98). In contrast, changes during treatment demonstrated substantially greater prognostic strength, with relative SMI and SFI decreases associated with HR of 1.17 and 1.11, respectively, consistent with previous studies by Chaunzwa et al. and Degens et al. in patients with NSCLC [2,11]. Thus, longitudinal assessment provides superior prognostic discrimination compared to baseline-focused approaches. Our results extend those from prior studies [2,11] to a broader population that also includes patients with RCC and melanoma, supporting the generalizability of this approach. Several mechanisms may underlie these associations. Loss of skeletal muscle and subcutaneous fat during treatment may reflect cancer-related cachexia, systemic inflammation, or decreased physiologic reserve, all of which are linked to poor treatment tolerance and adverse outcomes in immunotherapy [29,30]. Changes in body composition may also influence immune response through metabolic and endocrine pathways. Beyond its prognostic implications, automated CT-derived body composition analysis offers an objective and reproducible framework to quantify cancer-related metabolic remodeling and could serve as a standardized imaging biomarker for metabolic research in oncology.

The correlation structures of CT-derived body composition parameters highlight both redundancy and complementarity between tissue compartments. CT-derived fat compartments (SFI, VFI, IMFI) were highly interdependent, suggesting that multiple markers provide overlapping information; for example, VFI and IMFI largely capture similar aspects of adiposity. However, skeletal muscle showed weaker correlations with fat compartments, indicating that it represents a relatively distinct physiological domain that may provide complementary prognostic information when incorporated into multivariable models. These observations support incorporating both muscle and fat indices in risk stratification, as they reflect complementary biological processes relevant to patient outcomes.

This study is the first to utilize CompoCT, software developed at our tertiary cancer center, for evaluating CT-derived body composition parameters in a general oncology population. CompoCT provides fully automated segmentation and enables consistent, large-scale extraction of body composition metrics, which is essential for clinical implementation. While CompoCT offers additional subclassifications such as lower-density steatotic muscle, and higher-density lean muscle, these measures were not included in the current analysis due to variability in intravenous contrast use across scans, which can influence tissue density measurements. Therefore, fat and muscle attenuation values were excluded from our models, whereas subclassification metrics that are less sensitive to contrast, such as IMFI, were retained to preserve diagnostic value. Notably, longitudinal body composition assessment can be integrated into routine immunotherapy monitoring by extracting measurements from standard follow-up CT scans (e.g., 8–12 weeks post-baseline). Relative decreases in SMI or SFI exceeding 5% could trigger intensified metabolic support, nutritional intervention, or reassessment of treatment tolerance and prognosis. This approach is highly feasible, as it leverages imaging already acquired for tumor evaluation, adding minimal cost or workflow complexity.

Standardization of CT-derived body composition, including consistent terminology, segmentation methods, and reporting formats, is essential for advancing research and facilitating clinical translation [18]. We therefore reported changes using both absolute and relative metrics, and included age and sex in the multivariate analysis. Additionally, in the main analysis, we retained the actual time interval between scans without normalization, as body composition changes reflect underlying metabolic processes that are not linearly time-dependent during cancer treatment. Variability in compartment definitions across published studies and segmentation algorithms complicates direct comparisons; for example, some models include both intermuscular and intramuscular fat within the SMI layer, whereas others, including ours, analyze these compartments separately. CompoCT demonstrated robust performance in this clinical cohort, with expert quality assurance identifying L3 localization corrections in 5.7% of cases and segmentation exclusions in 6.2%. While most published automated body composition studies do not report such quality control metrics, transparent reporting of real-world algorithm performance is essential for clinical adoption and reproducibility.

The study has several limitations. First, its retrospective, single-center design, which focused on overall survival data, as progression-free survival data were not available. This design may introduce selection bias and limit the ability to establish causality. In addition, the cohort was heterogeneous and included three primary tumor types. While this heterogeneity introduces variability, it also enhances the real-world applicability and generalizability of the findings. Also, the cohort was characterized by heterogeneous imaging protocols, and the proportion of contrast-enhanced scans was not systematically recorded; however, analyses relied on segmentation-derived areas rather than attenuation-based metrics, reducing sensitivity to contrast-related variability. Another limitation was that clinical and laboratory confounders were available only for a subset of patients, limiting fully adjusted analyses to smaller sample sizes. Lastly, although our cohort was relatively large compared to most published studies in the field, the sample size remains modest compared to population-based datasets, and external validation in larger, independent cohorts is required to confirm our findings.

## 5. Conclusions

Longitudinal changes in CT-derived body composition parameters during immunotherapy were independently associated with overall survival in patients with solid tumors. Relative decreases in skeletal muscle and subcutaneous fat during treatment were stronger predictors of mortality than baseline values, highlighting the added prognostic value of incorporating these routinely available imaging metrics into clinical risk stratification and treatment monitoring. These findings highlight the potential of automated CT-based body composition analysis as a reproducible imaging biomarker for metabolic research and clinical monitoring in cancer.

## Figures and Tables

**Table 1:** Cohort characteristics: Demographics, treatments, and follow-up.

	Overall	NSCLC	RCC	Melanoma	*p* Value *
N	376	273 (72.6%)	55 (14.6%)	48 (12.8%)	
Age, mean (SD), years	66.4 (11.4)	67.1 (10.4)	63.3 (12.1)	65.6 (15.1)	0.074
Male, n (%)	253 (67.3%)	187 (68.5%)	40 (72.7%)	26 (54.2%)	0.097
Treatment regimen, n (%)					
Pembrolizumab	220 (58.5%)	199 (72.9%)	13 (23.6%)	8 (16.7%)	<0.001
Nivolumab	35 (9.3%)	16 (5.9%)	11 (20%)	8 (16.7%)	
Atezolizumab	13 (3.5%)	13 (4.8%)	0	0	
Durvalumab	1 (0.3%)	1 (0.4%)	0	0	
Ipilimumab + nivolumab	76 (20.2%)	26 (9.5%)	25 (45.5%)	25 (52.1%)	
Pembrolizumab + nivolumab	8 (2.1%)	4 (1.5%)	2 (3.6%)	2 (4.2%)	
Nivolumab + durvalumab	1 (0.3%)	1 (0.4%)	0	0	
Ipilimumab + pembrolizumab	2 (0.5%)	0	0	2 (4.2%)	
Pembrolizumab + atezolizumab	4 (1.1%)	4 (1.5%)	0	0	
Ipilimumab + pembrolizumab + nivolumab	12 (3.2%)	5 (1.8%)	4 (7.3%)	3 (6.3%)	
Ipilimumab + durvalumab + nivolumab	1 (0.3%)	1 (0.4%)	0	0	
Ipilimumab + pembrolizumab + atezolizumab	1 (0.3%)	1 (0.4%)	0	0	
Ipilimumab + atezolizumab + nivolumab	1 (0.3%)	1 (0.4%)	0	0	
Ipilimumab + pembrolizumab + atezolizumab + nivolumab	1 (0.3%)	1 (0.4%)	0	0	
Mortality during the study period, n (%)	220 (58.5%)	178 (65.2%)	22 (40.0%)	20 (41.7%)	<0.001
Follow-up time, median (min–max), months	21(2–95)	20 (2–95)	29 (3–86)	20 (4–85)	0.097
Interval between CT scans, median (min–max), days	100 (40–199)	98 (40–191)	104 (62–171)	108 (61–199)	0.021

CT, computerized tomography; NSCLC, non-small cell lung cancer; RCC, renal cell carcinoma. * *p* values were calculated using chi-square and ANOVA tests for categorical and continuous variables, respectively.

**Table 2:** Association between variables and overall mortality: multivariate analysis.

	HR	95% CI	*p* Value
Model 1			
Age (per year)	1.02	1–1.03	0.011
Sex (female vs. male)	1.06	0.80–1.41	0.697
Tumor type (NSCLC vs. melanoma and RCC)	1.66	1.18–2.33	0.003
Concordance	0.58		
Model 2			
Age (per year)	1.02	1–1.03	0.009
Sex (female vs. male)	0.93	0.70–1.24	0.641
Tumor type (NSCLC vs. melanoma and RCC)	1.78	1.27–2.5	<0.001
SFI relative decrease (5% units)	1.10	1.05–1.14	<0.001
SMI relative decrease (5% units)	1.10	1.01–1.21	0.031
Concordance	0.65		

CI, confidence interval; HR, hazard ratio; IMFI, intramuscular fat index; NSCLC, non-small cell lung cancer; RCC, renal cell carcinoma; SFI, subcutaneous fat index; SMI, skeletal muscle index.

## Data Availability

The datasets generated and analyzed in the current study are available from the corresponding author upon reasonable request.

## Supplementary Materials

The following supporting information can be downloaded at: https://www.mdpi.com/article/10.3390/cancers18020341/s1, Figure S1: Development and evaluation of the U-Net model for automatic localization of the mid-L3 vertebral level; Figure S2: Development and evaluation of the U-Net segmentation model for abdominal body composition analysis at the L3 vertebral level; Figure S3: Baseline CT timing distribution. Figure S4: Sensitivity analysis—Cox regression (pre-treatment baseline only); Figure S5: Scan-interval–normalized sensitivity analysis; Figure S6: Sensitivity analysis—3% decline cutoff; Figure S7: Sensitivity analysis—10% decline cutoff; Figure S8: Landmark Kaplan—Meier survival; Figure S9: Landmark Cox regression; Table S1: CT body composition distributions at baseline, follow-up, and the relative change from baseline. Table S2: Expanded multivariable Cox regression including clinical confounders Association between clinical variables, laboratory markers, and longitudinal body composition changes with overall mortality (subset analysis).
