# Supplementary material for: CT Body Composition Changes Predict Survival in Immunotherapy-Treated Cancer Patients: A Retrospective Cohort Study
Source: Cancers (Basel). 2026 Jan 21;18(2):341. doi: 10.3390/cancers18020341 (PMC12839126; doi:10.3390/cancers18020341)
Supplement: Supplementary file 1 [file cancers-18-00341-s001.zip › cancers-4099261-supplementary.pdf]

Supplementary Material

CT-Based Body Composition and Sarcopenia-related Laboratory Tests as Predictors of Survival in Immunotherapy-treated Cancer Patients

Shlomit Tamir[1,2], Hilla Vardi Behar,[3] Ronen Tal,[3] Ruthy Tal Jasper,[3] Mor Armoni, [1,2] Hadar Pratt Aloni,[3] Rotem Iris Orad,[2,3] Hillary Voet,[4] Eli Atar, [1,2] Ahuva Grubstein, [1,2] Salomon M Stemmer[2,3]*, Gal Markel[3,5]*

[1] Radiology Department, Rabin Medical Center-Beilinson Hospital, Petah Tikva, Israel
[2] Gray Faculty of Medicine and Health Sciences, Tel Aviv University, Tel Aviv, Israel
[3] Davidoff Cancer Center and Samueli Integrative Cancer Pioneering Institute, Rabin Medical Center-Beilinson Hospital, Petah Tikva, Israel
[4] Hebrew University of Jerusalem, Rehovot, Israel
[5] Department of Clinical Microbiology and Immunology, Gray Faculty of Medicine and Health Sciences, Tel Aviv, Israel

*These authors contributed equally to this work.

Corresponding author: Salomon M. Stemmer, Davidoff Cancer Center and Samueli Integrative Cancer Pioneering Institute, Rabin Medical Center, Jabotinsky St 39, Petah Tikva, 49100; Faculty of Medicine, Tel Aviv University, Tel Aviv, Israel. Tel. +972-50-4065467. Email: salomon.stemmer@gmail.com

Supplementary Methods

The CompoCT algorithm is a fully automated pipeline that quantifies body composition from three-dimensional computed tomography (CT) scans. It comprises two core deep learning models: (1) an L3 vertebral localization model that identifies the coordinates of the third lumbar vertebra, and (2) a slice segmentation model that delineates relevant tissue compartments on the corresponding axial slice. The overall workflow of the CompoCT pipeline is illustrated in Figure 1.

Model Architecture

Both the L3 localization and slice segmentation tasks were implemented using a shared two-dimensional U-Net–based convolutional neural network (CNN). This architecture employs an encoder–decoder design with skip connections that integrate low-level spatial features from early layers with high-level semantic information from deeper layers, enabling precise anatomical localization and segmentation.

The encoder progressively reduces spatial resolution through convolution and pooling operations to extract hierarchical features, while the decoder symmetrically upsamples and reconstructs detailed spatial information. The network receives a single-channel image (512 × 512 pixels) as input and outputs a pixel-wise segmentation map of the same size. A final 1x1 convolution with softmax activation produces the class probabilities for each pixel.

Mid-L3 vertebral localization

Dataset

The dataset initially comprised 866 abdominal CT scans acquired in the emergency department (ED) at Beilinson Hospital (Rabin Medical Center [RMC]) between January 2017 and November 2024. For each scan, the mid-L3 vertebra location in the full CT scan was selected by an expert with 4 years of experience in medical imaging. All mid-L3 selections were subsequently reviewed and validated by a fellowship trained abdominal radiologist with 17 years of experience. Following the exclusion of 38 scans due to incomplete spinal coverage or partial imaging series, the final dataset included 828 high-quality CT scans.

An external validation set was used to assess model generalizability. This set consisted of 172 abdominal CT scans acquired at Beilinson Hospital (RMC) during the same period but derived from a distinct patient population—individuals with a confirmed cancer diagnosis.

Preprocessing

Two-dimensional coronal maximum intensity projection (MIP) images were generated from each three-dimensional CT scan (Figure S1a). The pixel spacing was normalized to 1x1 mm^2 to ensure consistent input resolution for the L3 localization model. Each input MIP image was defined as 512x512 pixels. When the MIP height exceeded 512 pixels, the image was cropped to a fixed range of [−256, 256] from the center. For scans in which the MIP height was smaller than 512 pixels, zero-padding was applied to match the required input size. Intensity thresholds of 50 and 1500 Hounsfield units were used to emphasize bony and cartilaginous structures.

Results

A U-net model was trained, as described in section 1.1. The output was a segmentation map of identical dimensions containing two classes: (1) Mid-L3 vertebral segment – covering the entire row of pixels corresponding to the mid third lumbar vertebra in the MIP image. (2) Background – all remaining pixels.

The mean (SD) absolute localization error between the model-predicted and ground truth positions was 3.94 (7.0) mm. In the cross-validation dataset, 93.2% of images had an absolute error below 10 mm. Model performance was further evaluated on an independent external validation set comprising 172 CT scans from cancer patients (Figure S1c), in which 94.2% of cases achieved an absolute error below 10 mm.

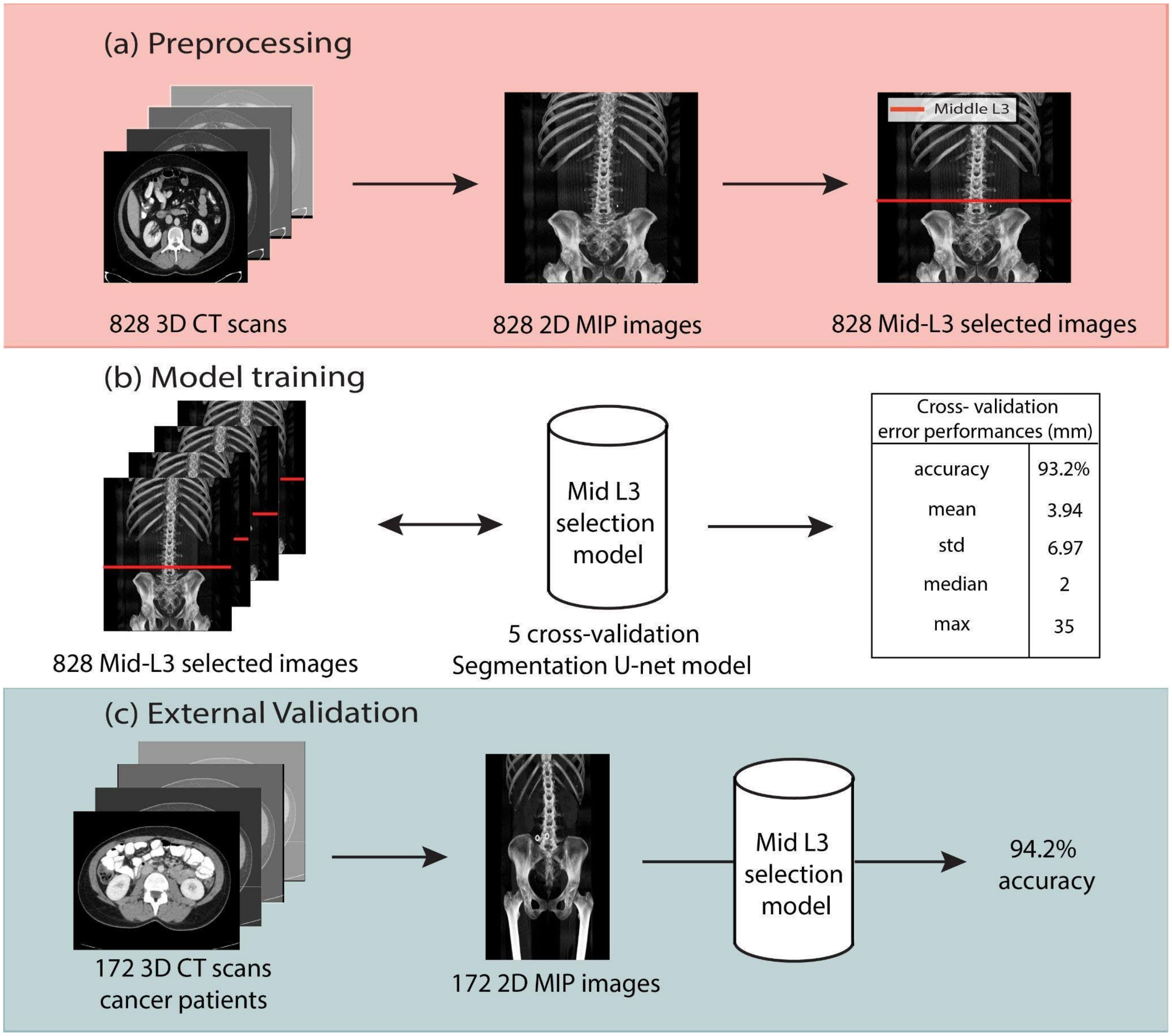

Figure S1. Development and evaluation of the U-Net model for automatic localization of the mid–L3 vertebral level.

(a) **Preprocessing:** A total of 828 three-dimensional abdominal CT scans were converted into two-dimensional coronal maximum intensity projection (MIP) images. Manual annotations were used to mark the mid–L3 vertebral level on each image, yielding a curated dataset of 828 labeled MIPs.

(b) **Model training:** A U-Net–based segmentation model was trained on the annotated dataset using five-fold cross-validation. Performance was evaluated by multiple error metrics: mean distance error = 3.94 mm, standard deviation = 6.97 mm, median = 2 mm, and maximum = 35 mm. Accurate localization was defined as an absolute error ≤10 mm, achieved in 93.2% of training images.

(c) **Model testing:** The trained model was applied to an independent external validation set of 172 CT scans from cancer patients. The corresponding MIP images were processed to predict the mid–L3 location, achieving 94.2% accuracy within the ≤10 mm threshold.

Slice Segmentation Model

Dataset

The training dataset comprised 671 abdominal CT scans acquired in the emergency department of Beilinson Hospital, Rabin Medical Center (RMC), between January 2017 and November 2024. For each scan, the axial slice at the L3 vertebral level was manually segmented by an expert with four years of experience in medical imaging. All segmentations were subsequently reviewed and validated by the Head of the Abdominal Radiology Department. After excluding 51 scans due to cropped anatomy or the presence of metallic implants, the final training dataset included 620 high-quality CT scans.

An external validation set was used to evaluate model generalizability. This set consisted of 125 abdominal CT scans acquired at Beilinson Hospital (RMC) during the same period, representing a distinct population of patients with a confirmed cancer diagnosis.

Results

A two-dimensional U-Net architecture model was trained to segment the axial L3 slice into anatomical compartments. The model input consisted of CT slices normalized to the standard abdominal window with 512 × 512 pixels, the default axial resolution. The model output is a 512 × 512 segmentation map comprising five classes: background, surrounding subcutaneous fat area (SFA), muscle layer, visceral fat area (VFA), and vertebra. Model architecture is described in section 1.1.

On the external validation set, the mean Dice similarity coefficients were 99.0% for background, 96.2% for vertebra, 93.0% for VFA, 92.8% for the muscle layer, and 96.0% for SFA. The overall development and evaluation workflow of the segmentation model is illustrated in Figure S2.

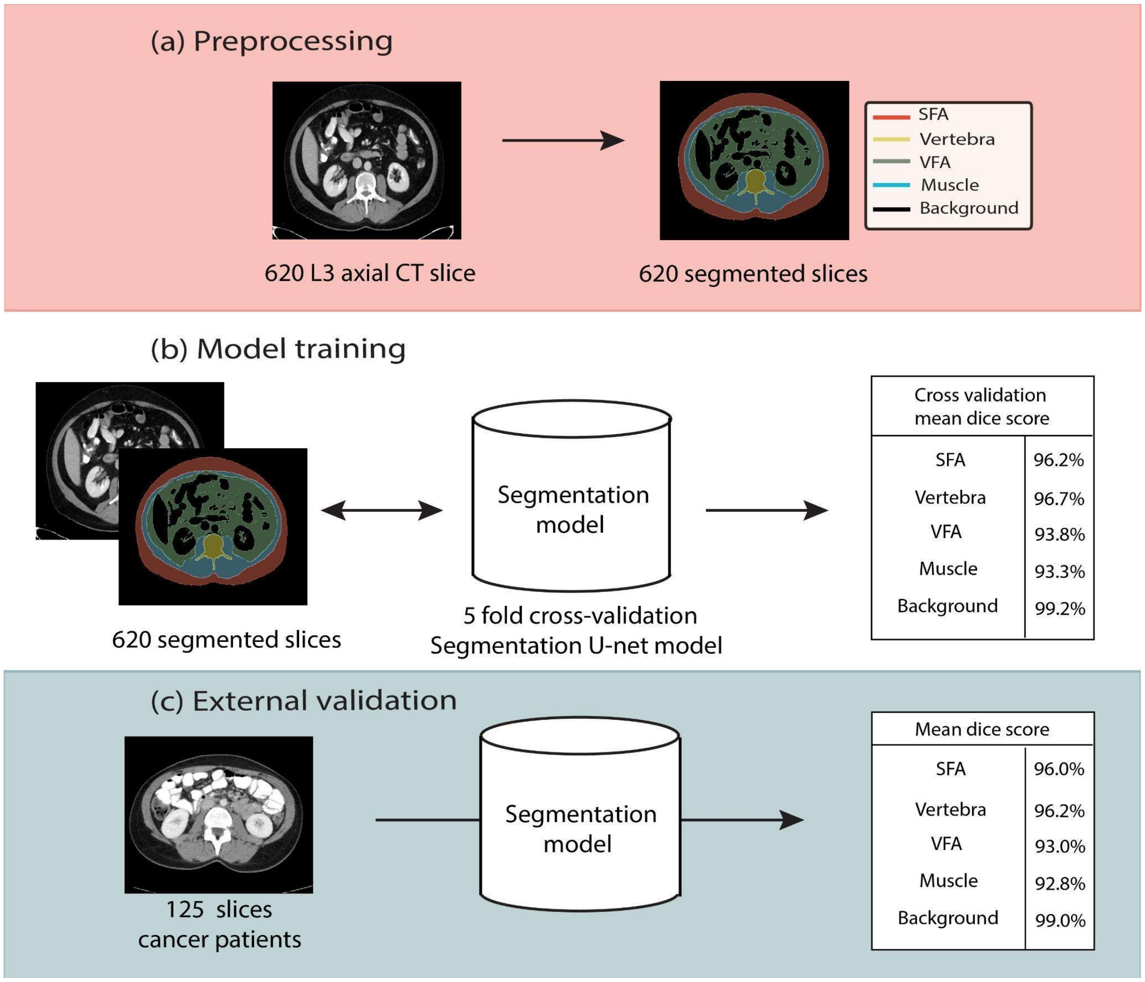

Figure S2. Development and evaluation of the U-Net segmentation model for abdominal body composition analysis at the L3 vertebral level.

(a) **Data:** A total of 620 axial CT slices at the L3 vertebral level were manually annotated for five anatomical compartments: subcutaneous adipose tissue (SFA, red), vertebra (yellow), visceral fat area (VFA, green), muscle (blue), and background (black).

(b) **Model training:** A U-Net–based segmentation model was trained on the annotated dataset using five-fold cross-validation. The model achieved high mean Dice similarity coefficients across all compartments: SFA = 96.2%, vertebra = 96.7%, VFA = 93.8%, muscle = 93.3%, and background = 99.2%.

(c) **Model evaluation:** The trained model was validated on an independent external validation set of 125 L3 slices from cancer patients, yielding comparable Dice scores: SFA = 96.0%, vertebra = 96.6%, VFA = 93.0%, muscle = 92.8%, and background = 99.0%.

Table S1. CT body composition distributions at baseline, follow-up, and the relative change from baseline.

Variable	No.	Median	Range	Mean	SD
SMI base (cm^2/m^2)	376	46.17	(25.88, 85.06)	47.19	9.23
SMI second (cm^2/m^2)	376	44.93	(25.44, 79.71)	45.27	8.80
SMI decrease (cm^2/m^2)	376	1.38	(-8.78, 17.08)	1.92	4.10
SMI relative decrease (unit per 5%)	376	0.65	(-4.61, 5.89)	0.73	1.68
SFI base (cm^2/m^2)	376	53.89	(8.5, 176.86)	61.16	32.51
SFI second (cm^2/m^2)	376	52.09	(3.34, 173.58)	58.44	32.27
SFI decrease (cm^2/m^2)	376	2.17	(-43.42, 49.42)	2.72	11.27
SFI relative decrease (unit per 5%)	376	0.78	(-18.88, 12.27)	0.76	3.97
VFI base (cm^2/m^2)	376	44.84	(2.73, 226.92)	52.46	34.91
VFI second (cm^2/m^2)	376	46.06	(1.61, 252.46)	51.94	35.21
VFI decrease (cm^2/m^2)	376	0.6	(-96.24, 43.79)	0.51	14.4
VFI relative decrease (unit per 5%)	376	0.24	(-117.29, 16.44)	-1.14	10.53
IMFI base (cm^2/m^2)	376	7.12	(0.64, 24.33)	7.6	4.09
IMFI second (cm^2/m^2)	376	7.44	(0.56, 22.91)	7.86	4.19
IMFI decrease (cm^2/m^2)	376	-0.18	(-12.67, 6.63)	-0.26	2.03
IMFI relative decrease (unit per 5%)	376	-0.74	(-65.17%, 12.64)	-1.4	6.54

IMFI, intramuscular fat index; SFI: Subcutaneous layer index; SMI, skeletal muscle index; VFI, visceral fat index.

Table S2. Expanded multivariable Cox regression including clinical confounders Association between clinical variables, laboratory markers, and longitudinal body composition changes with overall mortality (subset analysis). Model evaluated in a subset of patients with available ECOG and laboratory data (n = 118; 69 events)

	**HR**	**95% CI**	***P* value**
Age (per year)	1.01	0.99–1.03	0.46
Sex (female vs male)	1.01	0.59–1.73	0.96
Tumor type (NSCLC vs melanoma and RCC)	1.93	0.87–4.29	0.10
ECOG performance status (per unit)	1.52	1.06–2.16	0.02
Treatment regimen (combination vs single-agent)	0.92	0.45–1.88	0.81
Baseline albumin (per g/dL)	0.46	0.27–0.80	0.01
Baseline NLR	0.98	0.94–1.03	0.41
SFI relative decrease (per 5% units)	1.09	1.02–1.17	0.01
SMI relative decrease (per 5% units)	1.12	0.99–1.28	0.08
**Concordance (C-index)**	**0.70**		

CI, confidence interval; ECOG, Eastern Cooperative Oncology Group; HR, hazard ratio; NSCLC, non-small cell lung cancer; NLR, neutrophil-to-lymphocyte ratio; RCC, renal cell carcinoma; SFI: Subcutaneous layer index; SMI, skeletal muscle index.

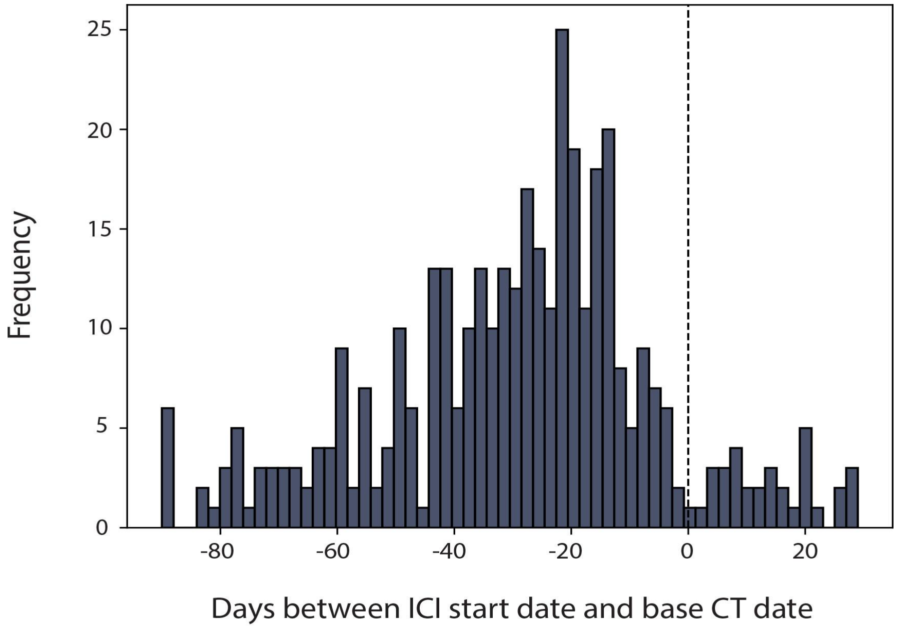

Figure S3. Baseline CT timing distribution. Distribution of baseline CT acquisition timing relative to initiation of immune checkpoint inhibitor therapy. Negative values indicate CTs obtained before treatment start; dashed line denotes treatment initiation.

(a) Univariate Cox models results

Variable	HR (95% CI)	p
Age (years)	1.02 (1.01-1.03)	0.002
NSCLC (vs. Melanoma and RCC)	1.64 (1.16-2.31)	0.005
Female (vs. Male)	0.98 (0.74-1.31)	0.911
Baseline SMI	0.99 (0.97-1.00)	0.063
Baseline SFI	1.00 (0.99-1.00)	0.450
Baseline VFI	1.00 (0.99-1.00)	0.739
Baseline IMFI	1.01 (0.98-1.05)	0.308
Follow- up SMI	0.97 (0.96-0.99)	0.001
Follow- up SFI	0.99 (0.99-1.00)	0.016
Follow- up VFI	1.00 (0.99-1.00)	0.115
Follow- up IMFI	0.99 (0.96-1.03)	0.775
Absolute decrease in SMI (per unit)	1.06 (1.03-1.10)	<0.001
Absolute decrease in SFI (per unit)	1.03 (1.02-1.04)	<0.001
Absolute decrease in VFI (per unit)	1.02 (1.01-1.03)	<0.001
Absolute decrease in IMFI (per unit)	1.11 (1.03-1.20)	0.009
Relative decrease in SMI (per 5% unit)	1.18 (1.08-1.29)	<0.001
Relative decrease in SFI (per 5% unit)	1.12 (1.08-1.17)	<0.001
Relative decrease in VFI (per 5% unit)	1.02 (1.00-1.04)	0.069
Relative decrease in IFI (per 5% unit)	1.02 (1.00-1.05)	0.071

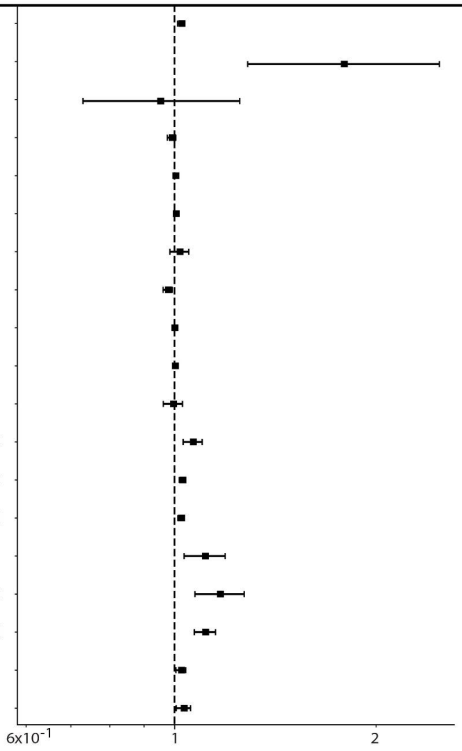

(b) Multivariate Cox models results

Variable	HR (95% CI)	p
Age (years)	1.02 (1.01-1.03)	0.003
NSCLC (vs. Melanoma and RCC)	1.61 (1.15-2.28)	0.006
Female (vs. Male)	0.91 (0.68-1.22)	0.512
Relative decrease in SMI (per 5% unit)	1.11 (1.01-1.22)	0.029
Relative decrease in SFI (per 5% unit)	1.11 (1.07-1.16)	<0.001

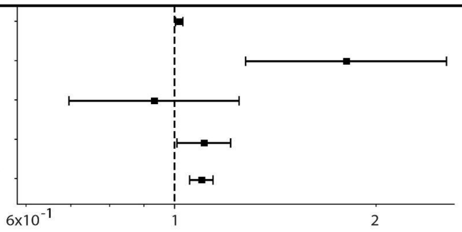

Figure S4. Sensitivity analysis – Cox regression (pre-treatment baseline only) . Univariate (a) and multivariable (b) Cox proportional hazards models from sensitivity analysis restricting baseline CT scans to the pre-treatment period only. Hazard ratios with 95% confidence intervals are shown.

(a) Univariate Cox models results

Variable	HR (95% CI)	p
Age (years)	1.02 (1.01-1.03)	0.004
Female (vs. Male)	1.01 (0.77-1.34)	0.924
NSCLC (vs. Melanoma and RCC)	1.80 (1.28-2.52)	0.001
Baseline SMI	0.99 (0.97-1.00)	0.043
Baseline SFI	1.00 (0.99-1.00)	0.250
Baseline VFI	1.00 (0.99-1.00)	0.492
Baseline IMFI	1.01 (0.98-1.05)	0.481
Follow- up SMI	0.97 (0.96-0.99)	<0.001
Follow- up SFI	0.99 (0.99-1.00)	0.007
Follow- up VFI	1.00 (0.99-1.00)	0.041
Follow- up IMFI	0.99 (0.96-1.02)	0.526
Absolute decrease in SMI (per unit)	1.17 (1.09-1.26)	<0.001
Absolute decrease in SFI (per unit)	1.06 (1.04-1.09)	<0.001
Absolute decrease in VFI (per unit)	1.04 (1.01-1.06)	0.003
Absolute decrease in IMFI (per unit)	1.23 (1.04-1.45)	0.014
Relative decrease in SMI (per 5% unit)	1.53 (1.28-1.84)	<0.001
Relative decrease in SFI (per 5% unit)	1.33 (1.22-1.46)	<0.001
Relative decrease in VFI (per 5% unit)	1.04 (1.00-1.08)	0.071
Relative decrease in IFI (per 5% unit)	1.04 (0.99-1.10)	0.137

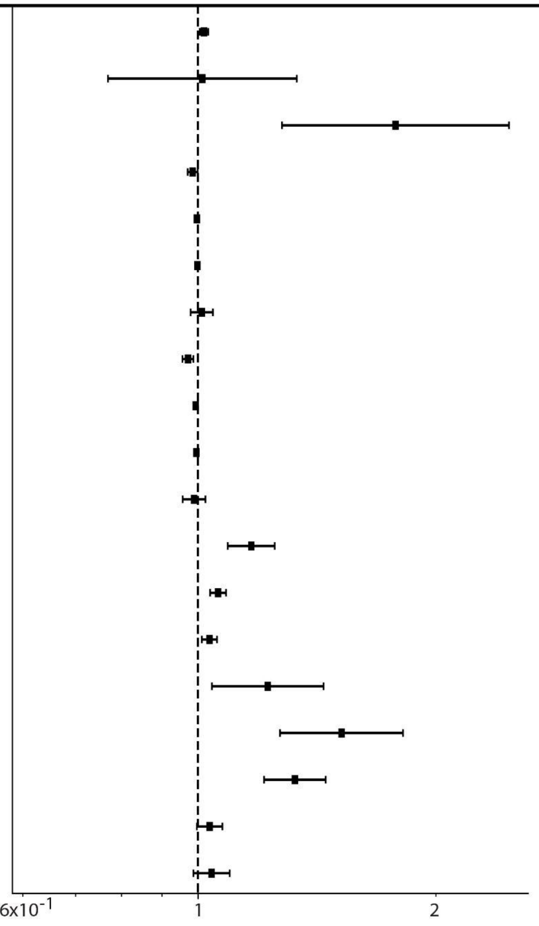

(b) Multivariate Cox models results

Variable	HR (95% CI)	p
Age (years)	1.02 (1.00-1.03)	0.009
NSCLC (vs. Melanoma and RCC)	1.77 (1.26-2.47)	0.001
Female (vs. Male)	0.96 (0.72-1.28)	0.789
Relative decrease in SFI (per 5% unit)	1.28 (1.17-1.41)	<0.001
Relative decrease in SMI (per 5% unit)	1.34 (1.10-1.62)	0.003

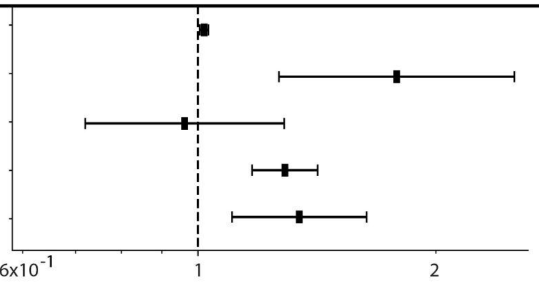

Figure S5. Scan-interval–normalized sensitivity analysis. Cox proportional hazards models using longitudinal body composition changes normalized by scan interval (relative percentage change per 30 days). Univariate (a) and multivariable (b) hazard ratios with 95% confidence intervals are shown

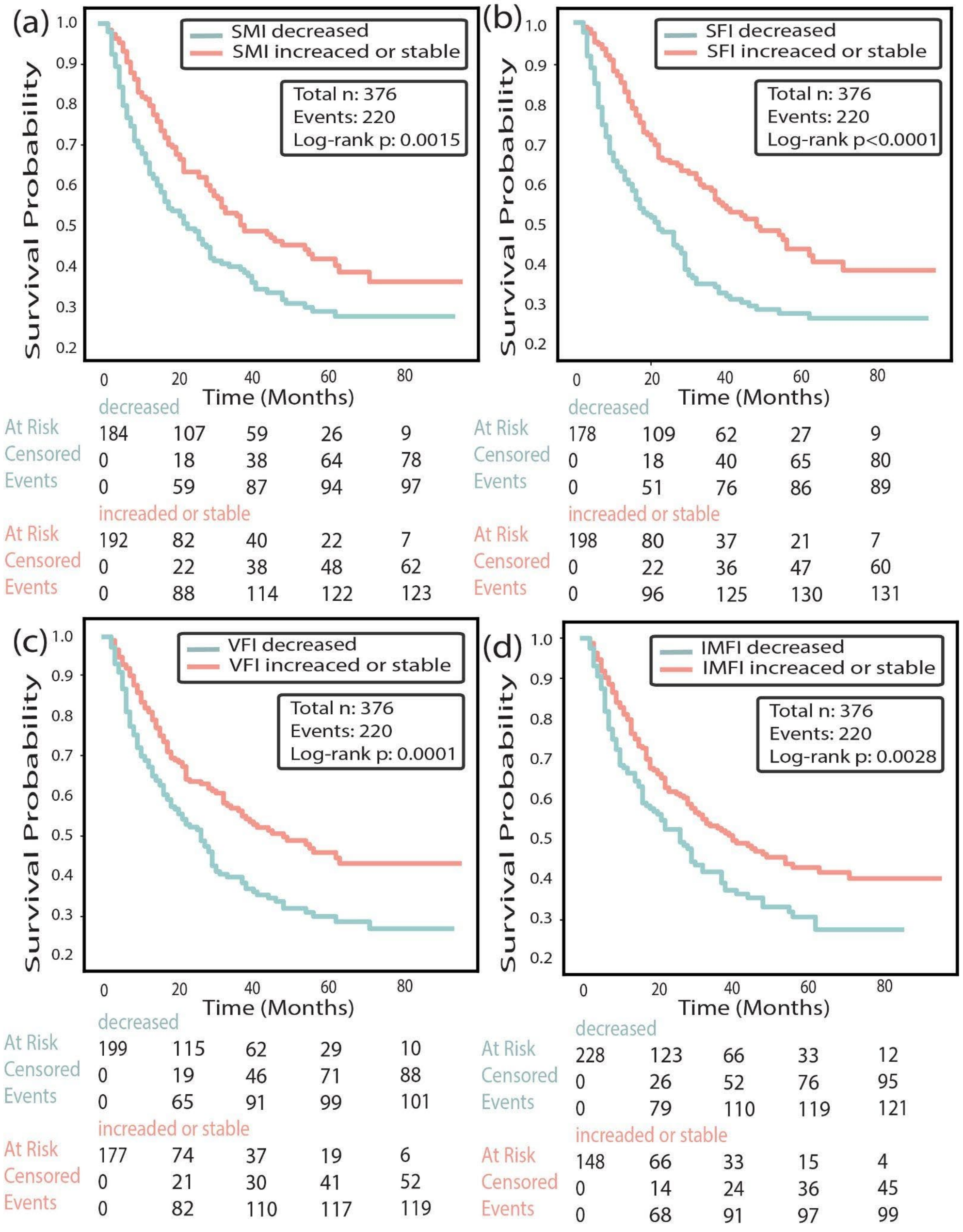

Figure S6. Sensitivity analysis – 3% decline cutoff. Kaplan–Meier overall survival curves stratified by ≥3% relative decline in body composition indices (SMI, SFI, VFI, and IMFI). Log-rank p-values are shown.

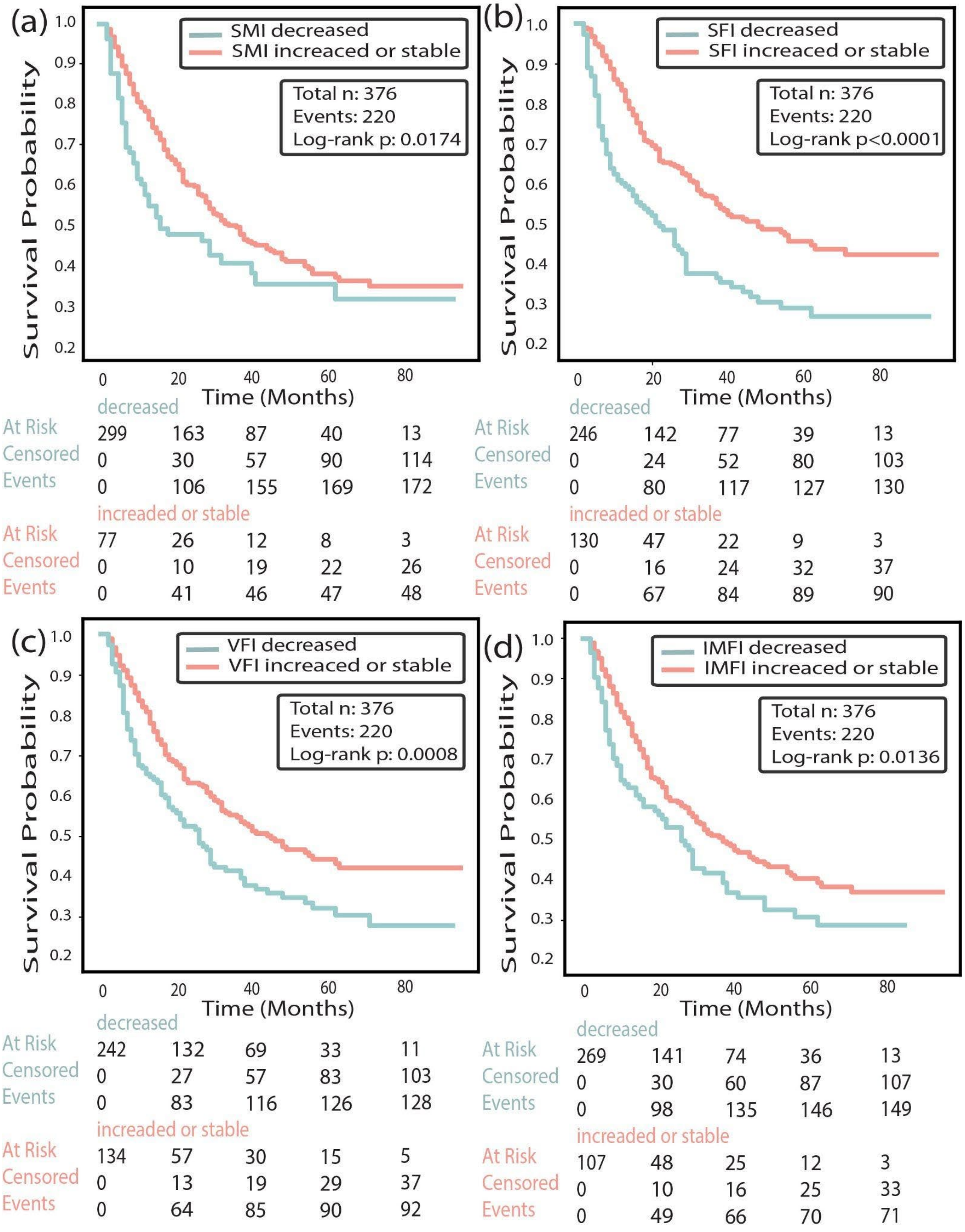

Figure S7 Sensitivity analysis – 10% decline cutoff. Kaplan–Meier overall survival curves stratified by ≥10% relative decline in body composition indices (SMI, SFI, VFI, and IMFI). Log-rank p-values are shown.

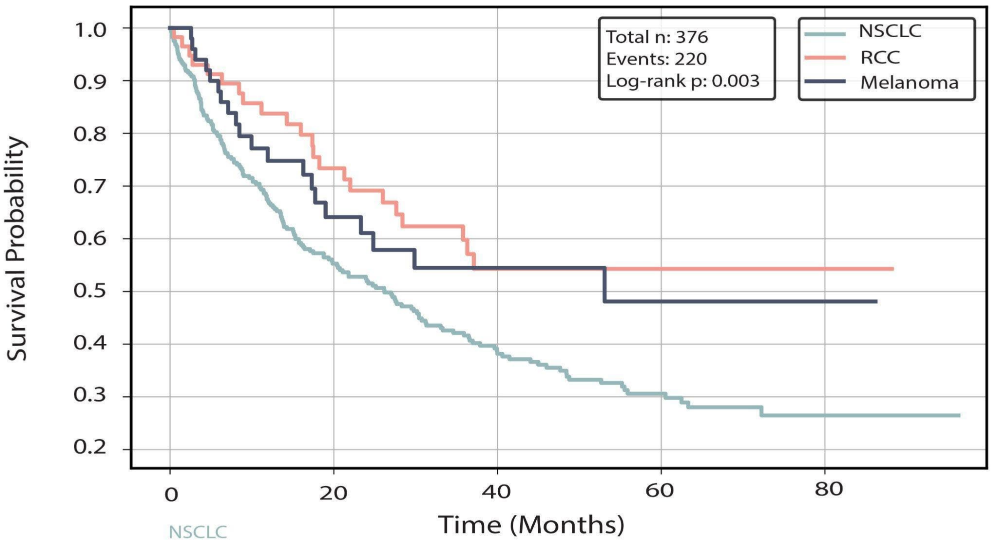

	0	20	40	60	80
**NSCLC**					
At Risk	273	124	67	30	9
Censored	0	24	44	68	86
Events	0	125	162	175	178
**RCC**					
At Risk	55	33	18	9	3
Censored	0	8	15	24	30
Events	0	14	22	22	22
**Melanoma**					
At Risk	48	21	11	6	4
Censored	0	11	18	22	24
Events	0	16	19	20	20

Figure S8. Landmark Kaplan–Meier survival. Kaplan–Meier overall survival curves stratified by tumor type (NSCLC, RCC, melanoma). Time zero corresponds to the follow-up CT scan (landmark). Log-rank p-values and numbers at risk are shown.

(a) Univariate Cox models results

Variable	HR (95% CI)	p
Age (years)	1.02 (1.01-1.03)	0.004
NSCLC (vs. Melanoma and RCC)	1.79 (1.28-2.50)	<0.001
Female (vs. Male)	1.01 (0.76-1.34)	0.939
Baseline SMI	0.99 (0.97-1.00)	0.044
Baseline SFI	1.00 (0.99-1.00)	0.257
Baseline VFI	1.00 (0.99-1.00)	0.500
Baseline IMFI	1.01 (0.98-1.05)	0.463
Follow- up SMI	0.97 (0.96-0.99)	<0.001
Follow- up SFI	0.99 (0.99-1.00)	0.008
Follow- up VFI	1.00 (0.99-1.00)	0.041
Follow- up IMFI	0.99 (0.96-1.02)	0.539
Absolute decrease in SMI (per unit)	1.06 (1.03-1.10)	<0.001
Absolute decrease in SFI (per unit)	1.02 (1.01-1.03)	<0.001
Absolute decrease in VFI (per unit)	1.02 (1.01-1.03)	<0.001
Absolute decrease in IMFI (per unit)	1.11 (1.03-1.19)	0.006
Relative decrease in SMI (per 5% unit)	1.17 (1.07-1.27)	<0.001
Relative decrease in SFI (per 5% unit)	1.11 (1.07-1.15)	<0.001
Relative decrease in VFI (per 5% unit)	1.02 (1.00-1.04)	0.054
Relative decrease in IFI (per 5% unit)	1.03 (1.00-1.05)	0.042

(b) Multivariate Cox models results

Variable	HR (95% CI)	p
Age (years)	1.02 (1.00-1.03)	0.009
NSCLC (vs. Melanoma and RCC)	1.76 (1.26-2.47)	0.001
Female (vs. Male)	0.93 (0.70-1.24)	0.642
Relative decrease in SMI (per 5% unit)	1.10 (1.01-1.21)	0.032
Relative decrease in SFI (per 5% unit)	1.10 (1.05-1.14)	<0.001

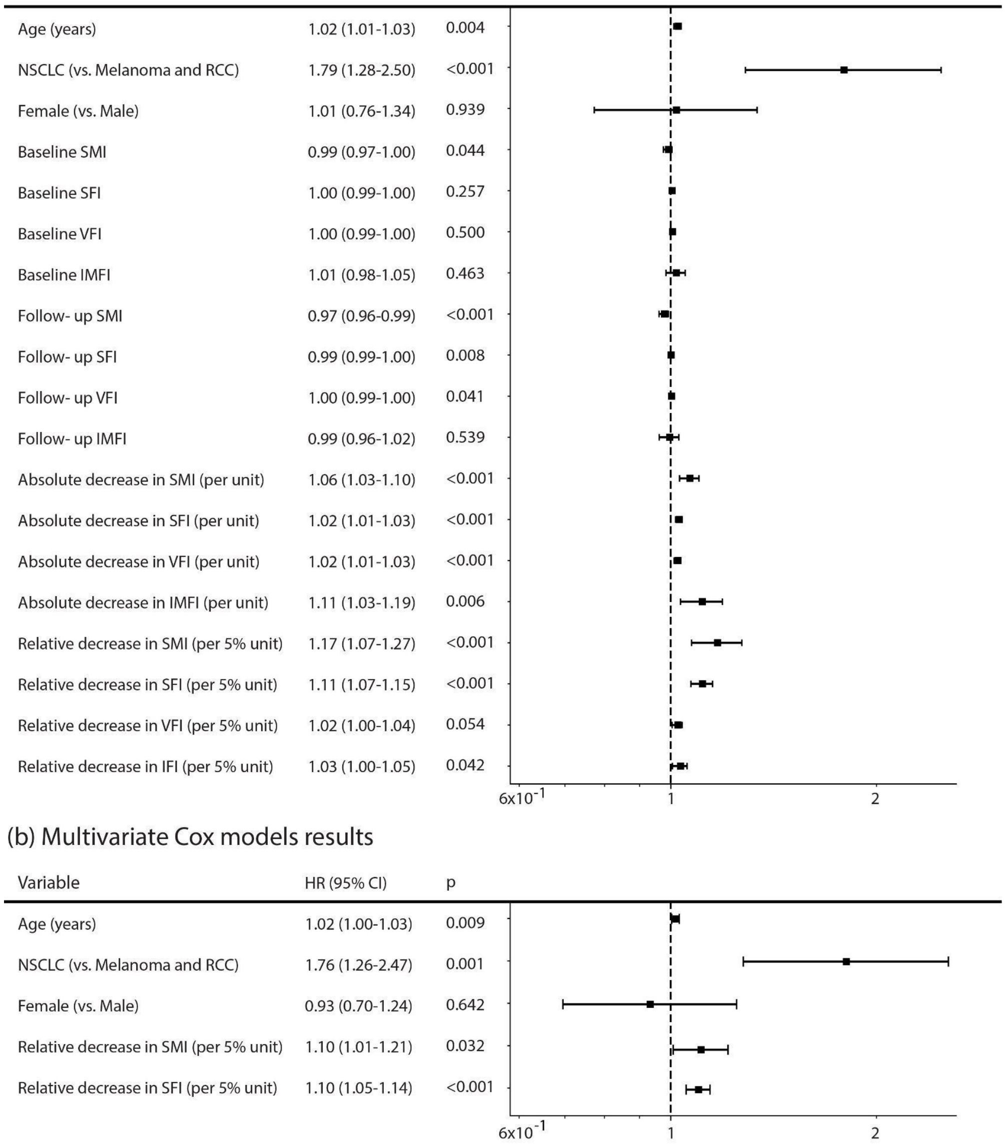

Figure S9. Landmark Cox regression. Cox proportional hazards models from landmark analysis evaluating associations between clinical variables and longitudinal body composition changes with overall survival. Survival time was calculated from the follow-up CT scan (landmark). Hazard ratios with 95% confidence intervals are shown for the univariate (a) and multivariate (b) analyses.
